# Identification of aging-related biomarkers for intervertebral disc degeneration in whole blood samples based on bioinformatics and machine learning

**DOI:** 10.3389/fimmu.2025.1565945

**Published:** 2025-04-15

**Authors:** Zi-hang Li, Shi-pian Li, Ya-hao Li, Yu-cheng Wang, Zhen-yu Tang, Kai-yang Xu, Xiao-rong Li, Zhen Tan, Jiao-yi Pan, Jin-tao Liu, Hong Jiang, Zhi-jia Ma, Yu-xiang Dai, Peng-fei Yu

**Affiliations:** ^1^ Department of Orthopedics, Suzhou TCM Hospital Affiliated to Nanjing University of Chinese Medicine, Suzhou, Jiangsu, China; ^2^ Spine Institute, Longhua Hospital, Shanghai University of Traditional Chinese Medicine, Shanghai, China; ^3^ Key Laboratory of Theory and Therapy of Muscles and Bones, Ministry of Education, Shanghai University of Traditional Chinese Medicine, Shanghai, China; ^4^ Department of Orthopedics, Wenzhou Hospital of Integrated Traditional Chinese and Western Medicine, Wenzhou, Zhejiang, China

**Keywords:** intervertebral disc degeneration, aging-related biomarkers, bioinformatics, machine learning, immune infiltration analysis, whole blood samples

## Abstract

**Introduction:**

Aging is characterized by gradual structural and functional changes in the body over time, with intervertebral disc degeneration (IVDD) representing a key manifestation of spinal aging and a major contributor to low back pain (LBP).

**Methods:**

This study utilized bioinformatics and machine learning approaches to identify aging-related biomarkers associated with IVDD in whole blood samples. By analyzing GEO datasets alongside aging-related databases such as GeneCards, HAGR, and AgeAnno, we identified 15 aging-related differentially expressed genes (AIDEGs). Correlation and immune infiltration analyses were conducted on these AIDEGs, and diagnostic models were developed using WGCNA, logistic regression, random forest, support vector machine, k-nearest neighbors, and LASSO regression to identify key genes.

**Results:**

Among these, *FCGR1A*, *CBS*, and *FASLG* emerged as significant biomarkers with strong predictive capabilities for IVDD. Further exploration of biological pathways involving AIDEGs provided insights into their potential roles in IVDD pathogenesis. To further validate these findings, we collected human blood specimens and conducted *in vitro* experiments. ELISA assays confirmed that CBS and FASLG are crucial biomarkers of IVDD, with distinct expression patterns in patients with moderate versus severe degeneration.

**Discussion:**

These results highlight the diagnostic potential of AIDEGs and provide a new perspective for early intervention and treatment strategies in IVDD.

## 1 Introduction

Low back pain (LBP) is a prevalent global public health issue that severely impacts patients’ quality of life. Intervertebral disc degeneration (IVDD) is a leading cause of LBP (1), accounting for a significant portion of disability-adjusted life years related to musculoskeletal conditions. The pathogenesis of IVDD involves multiple factors, including cell death, immune activation, inflammation, and mechanical stress.

Aging, as an inevitable consequence over time, is often accompanied by changes in various biological processes, such as cellular senescence, mitochondrial dysfunction, and chronic inflammation (2), which further influence the progression of IVDD. In older adults, IVDD is a common form of lumbar degenerative disease, posing significant physical and psychological harm to this population (3).

Despite advances in understanding the pathological mechanisms of IVDD, early diagnostic biomarkers that can accurately reflect disease progression remain lacking. Current studies have predominantly focused on localized intervertebral disc tissues, which are challenging to access and often require invasive procedures. This has limited the development of diagnostic and therapeutic strategies applicable to broader clinical settings. In contrast, whole blood samples represent a more accessible and less invasive source for biomarker identification, enabling the detection of systemic molecular changes associated with IVDD progression.

Recent studies have identified aging-related genes within the nucleus pulposus (NP) tissue of degenerated discs (4). However, the relationship between aging-related genes and IVDD in whole blood samples remains largely unexplored. Given the immune-privileged nature of intervertebral discs, the rupture or degeneration of disc tissue can activate systemic immune responses, resulting in the release of biomarkers into the peripheral blood (5, 6). Therefore, by detecting aging-related genes and immune factors in whole blood, it is possible to indirectly reflect the biological changes occurring during the process of IVDD. This underscores the potential utility of blood-based aging-related biomarkers in reflecting biological changes during IVDD progression and informing early intervention strategies.

Since its emergence, bioinformatics has been extensively applied in the analysis of public biological data, facilitating the identification of potential disease targets and supporting the development of novel therapeutics (7). At the same time, the expansion and increasing complexity of biological data have driven the application of machine learning in biology, such as the use of various algorithms to establish predictive models and identify potential key genes (8).

To address these gaps, this study aimed to identify aging-related biomarkers of IVDD using whole blood samples. By combining bioinformatics and machine learning approaches, we sought to uncover aging-related differentially expressed genes (AIDEGs) and evaluate their potential as diagnostic markers. Furthermore, we validated our findings through *in vitro* experiments on human specimens, providing a comprehensive framework to understand the molecular mechanisms underlying IVDD. This approach not only advances the identification of non-invasive biomarkers, but also lays the groundwork for future therapeutic strategies targeting IVDD.

## 2 Materials and methods

### 2.1 Data source and processing

We collected data from GSE150408 and GSE124272 in the Gene Expression Omnibus (GEO) database, both of which include gene expression data from whole blood samples of patients with IVDD. The GSE150408 dataset contained 59 samples; after excluding 24 treatment samples, we retained 17 healthy and 17 IVDD samples. The GSE124272 dataset included eight healthy and eight IVDD samples. The average age was 23 years in the healthy group and 40 years in the IVDD group. All included samples were free from other spinal, cardiovascular, metabolic, rheumatic, immune, or infectious diseases, aside from IVDD (9). After obtaining platform annotation files, we annotated probes for each dataset. We then performed normalization of expression profiles within each dataset using the normalizeBetweenArrays function from the “limma” package. Next, we merged GSE124272 and GSE150408 and applied the ComBat function from the “sva” package to eliminate batch effects between the two datasets.

### 2.2 Differentially expressed gene (DEG) extraction

We used the “limma” package to identify DEGs. First, we selected genes with non-zero expression in over 75% of the samples. DEGs were then filtered based on criteria of |logFC| > 0 and p < 0.05, categorizing them into upregulated, downregulated, and non-significantly changed genes. We visualized the DEGs with a volcano plot using the “ggplot2” package.

### 2.3 Acquisition of aging-related genes

We obtained aging-related genes (ARGs) from GeneCards and selected those with a relevance score > 7. Additionally, we incorporated ARGs from the Human Ageing Genomic Resources (HAGR) database based on the study by Zhou et al. (10), and retrieved ARGs from the AgeAnno database (11), filtering for genes sourced from “Blood.” We removed duplicates among the genes from these three sources and included the consolidated set of ARGs for further analysis.

### 2.4 Biological pathway enrichment analysis

We uploaded the obtained DEGs and ARGs to the Xiantao Academic Platform (https://www.xiantaozi.com) to generate a Venn diagram online, identifying intersecting genes as AIDEGs for IVDD. Next, we imported AIDEGs into Sangerbox 3.0 (http://www.sangerbox.com/) for gene ontology (GO) and Kyoto Encyclopedia of Genes and Genomes (KEGG) enrichment analyses, setting the criteria as Benjamini–Hochberg adjusted FDR < 0.1 and p < 0.05 (12). For GSEA enrichment analysis, we used the “clusterProfiler” package on AIDEGs, setting the threshold at p < 0.05. The gene set “c2.all.v2024.1.Hs.entrez” was downloaded from the Msigdb database, with a minimum gene set size of 10 and a maximum of 500. The “gseaplot2” function was used to visualize the results.

### 2.5 Weighted gene co-expression network analysis

For the merged dataset, we performed weighted gene co-expression network analysis (WGCNA) using the applicable package. First, we loaded the gene expression matrix and used the “pickSoftThreshold” function to determine the optimal soft threshold to construct a scale-free network. We then validated the scale-free properties of the network. The “adjacency” function was used to generate the adjacency matrix, which was transformed into a topological overlap matrix (TOM) to measure gene connectivity within the network. We calculated the dissimilarity (1 - TOM) and performed hierarchical clustering based on this to generate a gene clustering dendrogram. The “cutreeDynamic” function was used for dynamic tree cutting, with a minimum of 30 genes per module, and the clustering results were visualized.

To reduce network complexity, we merged modules with a similarity greater than 0.75 and generated a clustering result plot. Finally, we assessed the correlation between gene modules and clinical phenotypes, identifying the modules most strongly associated with IVDD and the genes contained within these modules.

### 2.6 Hub genes identification and gene correlation analysis

We imported the AIDEGs and module genes obtained from WGCNA into the Xiantao Academic Platform to create a Venn diagram, identifying the intersecting genes as hub genes. We then used the “pheatmap” package in R to generate a heatmap of gene expression. Differential analysis was conducted using the “ggpubr” package to create grouped comparison plots. Finally, a correlation analysis was performed using the “PerformanceAnalytics” package.

### 2.7 Immune infiltration analysis and protein–protein interaction (PPI) network construction

We performed immune infiltration analysis on the merged dataset using the “CIBERSORT” package and visualized the results to observe the expression of immune-related cells in the whole blood samples of patients with IVDD. Additionally, we conducted Spearman correlation analysis between the hub genes and immune cells to explore their relationships. Finally, we imported all hub genes into String (www.string-db.org) to construct a PPI network, selecting Homo sapiens with high confidence.

### 2.8 Construction and optimization of the diagnostic model

The samples were randomly divided into a training set and a validation set, with the training set comprising 70% of the total samples. To ensure the comparability between the two datasets, we compared the gene expression profiles of patients in both groups. No statistically significant differences observed in gene expression between the two groups indicated that the datasets were suitable for model construction and validation.

### 2.9 Logistic regression

For all the hub genes, we performed univariate logistic regression analysis and included genes with a p-value < 0.2 in the multivariate logistic regression analysis. The stepwise regression method was used to select key variables. To visualize the performance of the predictive model, we generated calibration curves, decision curve analysis (DCA) plots, and nomograms. We assessed the model’s discrimination and calibration ability using the C-index and Hosmer–Lemeshow test. Finally, we plotted the ROC curve to evaluate the diagnostic performance of the key genes.

### 2.10 Random forest

We constructed a random forest (RF) classification prediction model using the expression profile data from the training set. An out-of-bag (OOB) error curve was generated to visualize how the error rate changes as the number of trees increases in the model. The “varImpPlot” function was used to create a feature importance plot to assess the contribution of each gene in the model. Finally, the validation set was used to evaluate the predictive ability of the model, and the area under the curve (AUC) value was calculated to assess the performance of the final model.

### 2.11 Support vector machine (SVM)-RFE feature selection and SVM model construction

To optimize the prediction performance and generalization ability of the SVM model, we first used the SVM-RFE method to select feature genes. We downloaded the “msvmRFE” package by John Colby and applied 10-fold cross-validation to extract feature genes. Next, we used the “FeatSweep.wrap” function to perform iterative testing on the 15 hub genes to evaluate the impact of different feature quantities on the model’s performance. Finally, we calculated the error values for each feature selection step, plotted error curves, and accuracy curves, and selected the feature genes based on these results.

After feature gene selection, we processed the training and validation sets. Based on the training set, we used a linear kernel SVM model, optimizing the model by adjusting different cost parameters to select the best model. The training set was input into the optimal SVM model to obtain prediction results, which were compared with the actual groupings. A box plot was drawn to visualize the differences and perform significance testing. Next, the validation set was input into the optimal SVM model to obtain prediction results, and a box plot was drawn to assess the model’s performance. Finally, ROC curves for both the training and validation sets were plotted, and the AUC value was calculated to evaluate the model’s discrimination and generalization ability.

### 2.12 K-nearest neighbor (KNN) algorithm

We employed KNN algorithms to conduct predictions on the validation set. By computing the KNN of each sample in the validation set and determining their class labels, the prediction outcomes were obtained. To enhance the model’s robustness and generalizability, five-fold cross-validation was employed. Subsequently, a confusion matrix was generated to analyze the specific performance of the classification results, encompassing indicators such as accuracy, precision, etc., to comprehensively assess the classification efficacy of the model. Finally, based on the prediction results of the validation set, an ROC curve was plotted and the AUC value was calculated to evaluate the discriminatory capacity of the model.

### 2.13 Least absolute shrinkage and selection operator (LASSO) regression

A LASSO regression model was employed with 10-fold cross-validation, and a cross-validation curve for λ was generated to illustrate the model’s performance across different λ values, facilitating the selection of the optimal penalty parameter. Additionally, a LASSO coefficient trajectory plot was constructed, providing a visual representation of the changes in regression coefficients for individual genes at varying λ values. Finally, by analyzing the regression results, genes with non-zero regression coefficients were identified and extracted as key genes.

### 2.14 Ethical statement

This study involving human participants was approved by the Medical Ethics Committee of Suzhou Traditional Chinese Medicine Hospital (Approval No. 2023 LUNYANPI 020) and adheres to the ethical principles outlined in the Ministry of Health’s “Ethical Review Measures for Life Science and Medical Research Involving Humans (2023),” SFDA’s “Good Clinical Practice for Drug Trials (2020)”, “Regulations on Clinical Trials of Medical Devices (2016)”, WMA’s “Declaration of Helsinki”, and CIOMS’ “International Ethical Guidelines for Biomedical Research Involving Human Subjects.” All participants provided written informed consent prior to inclusion in the study, and measures were taken to ensure confidentiality and anonymity of their data.

### 2.15 Enzyme-linked immunosorbent assay (ELISA)

An ELISA was performed to assess the protein expression levels in human serum according to standard protocols. Morning fasting blood samples were collected from hospitalized patients with lumbar disc herniation in the Department of Orthopedics, Suzhou Hospital of Traditional Chinese Medicine. Patients were instructed to fast from food and water starting at 10:00 PM the previous day, and venous blood samples were drawn the following morning at 7:00 AM after an overnight fast. All included patients did not have any other spinal disorders (e.g., lumbar spondylolisthesis, spinal fractures) or chronic diseases (e.g., hypertension, diabetes). Additionally, patients included in the study had not received anti-inflammatory drugs, steroids, or other pharmaceutical interventions for at least 1 week prior to treatment. The serum samples were allowed to stand at 25°C ± 3°C for 15 minutes, followed by centrifugation at 15,000 rpm for 15 minutes. The resulting supernatant (SN) was collected for ELISA analysis. The levels of *Cystathionine-beta-synthase (CBS)* (CUSABIO, China, CSB-E13314h), *Fas/TNFRSF6/CD95* (MULTI SCIENCES, China, EK1F01-AW1), and *CD64* (CAMILO BIOLOGICAL, China, 2H-KMLJh312569) were quantified using corresponding ELISA kits, with procedures carried out strictly according to the manufacturer’s instructions to ensure accuracy and reproducibility.

### 2.16 Statistical analysis

All statistical analyses were performed using R (version 4.4.1). The differences in hub genes between the groups and the immune infiltration analysis were assessed using the Wilcoxon test. The accuracy of the model was evaluated by the AUC. ELISA results were analyzed using GraphPad Prism 9.10. Comparisons between the CON, M-IVDD, and S-IVDD groups were conducted using either one-way ANOVA or Brown–Forsythe and Welch’s ANOVA, depending on the homogeneity of variance. Furthermore, Tukey’s multiple comparisons and unpaired t-tests with Welch’s correction for pairwise group comparisons were used accordingly. A p-value < 0.05 was considered statistically significant, with *P < 0.05, **P < 0.01, and ***P < 0.001.

## 3 Results

### 3.1 Microarray data

**Figure 1:**
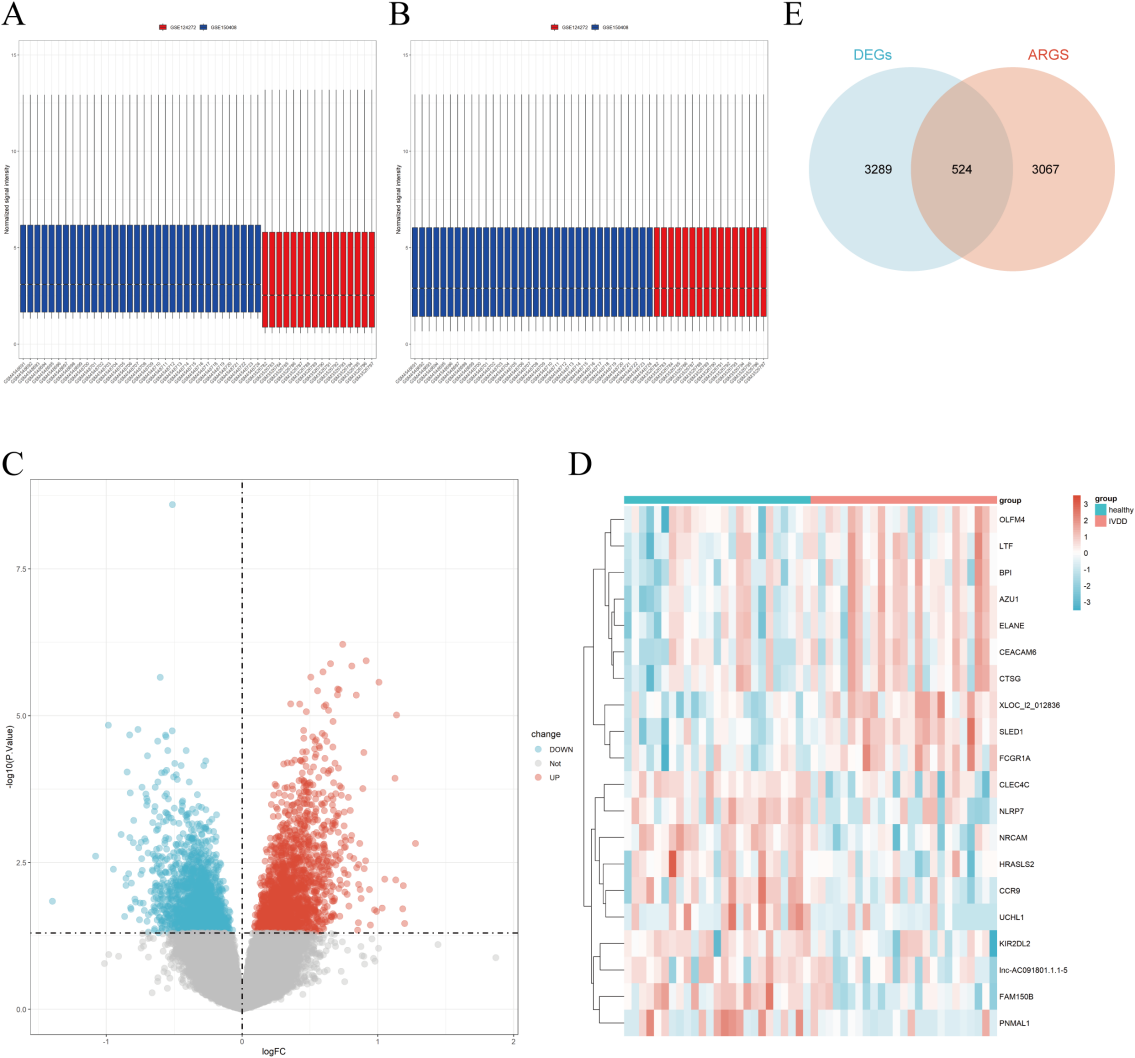
GSE data integration and analysis. **(A)** Results before batch effect removal for GSE150408 and GSE124272. **(B)** Results after batch effect removal for GSE150408 and GSE124272. **(C)** Volcano plot of DEGs. **(D)** Heatmap of top 10 upregulated and downregulated DEGs. **(E)** Intersection of DEGs with aging-related genes, resulting in aging-related DEGs (AIDEGs).

Microarray datasets GSE150408 and GSE124272 were obtained from the GEO database, with no logarithmic transformation required. After merging GSE124272 and GSE150408, batch effects were removed (**Figures 1A, B**). A total of 3,813 DEGs were identified, including 1,875 upregulated and 1,938 downregulated genes. Visualizations included a volcano plot and heatmaps for the top 10 upregulated and downregulated DEGs (**Figures 1C, D**). Subsequently, genes obtained from GeneCards, AgeAnno, and HARG were deduplicated and intersected with the DEGs, resulting in 524 aging-related DEGs (AIDEGs), as depicted in **Figure 1E**.

### 3.2 Enrichment analysis results

**Figure 2:**
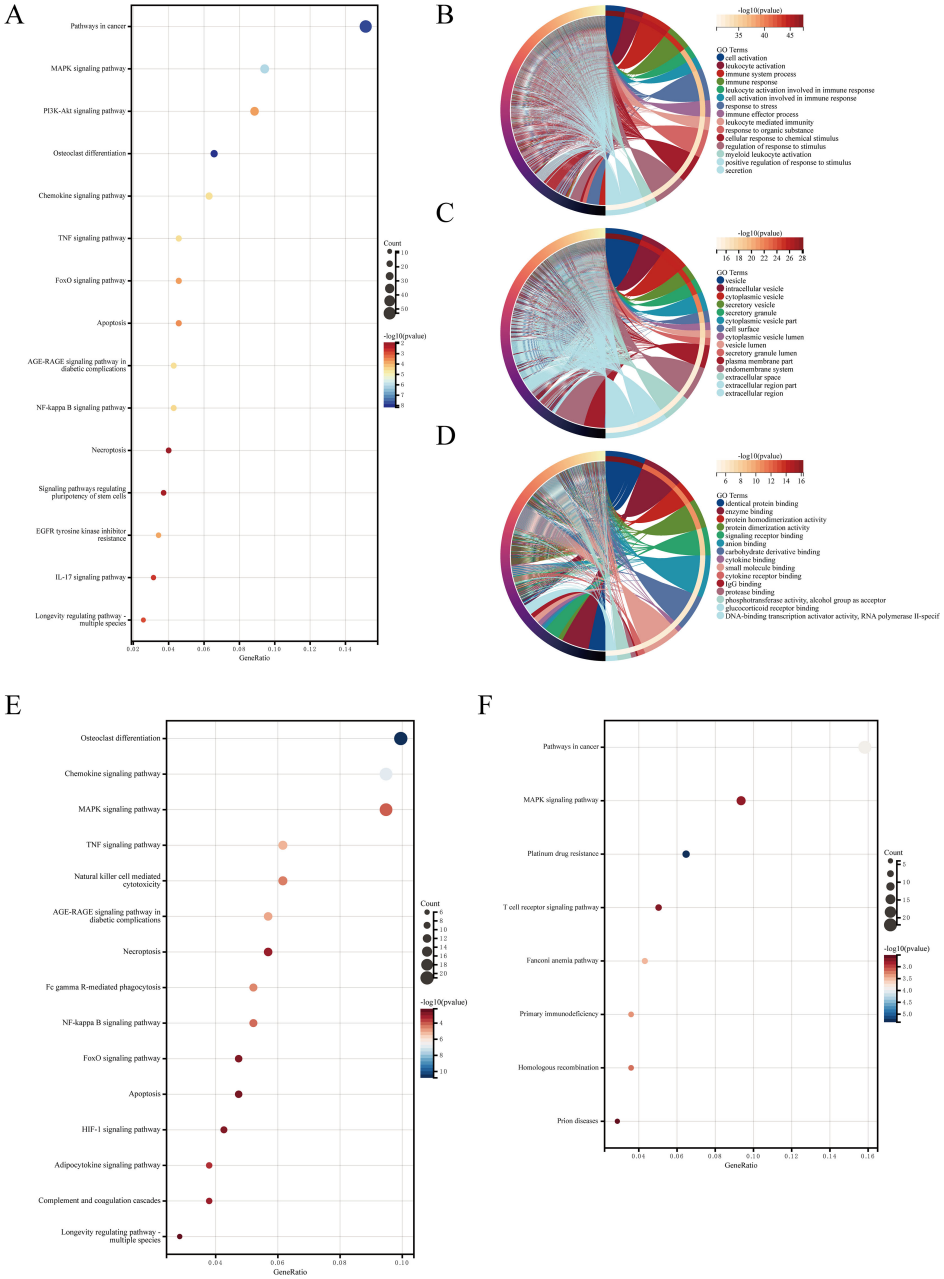
Enrichment analysis results. **(A)** KEGG pathway analysis results. **(B)** GO analysis of biological processes. **(C)** GO analysis of cellular components. **(D)** GO analysis of molecular functions. **(E)** KEGG pathway analysis for upregulated AIDEGs. **(F)** KEGG pathway analysis for downregulated AIDEGs.

**Figure 3:**
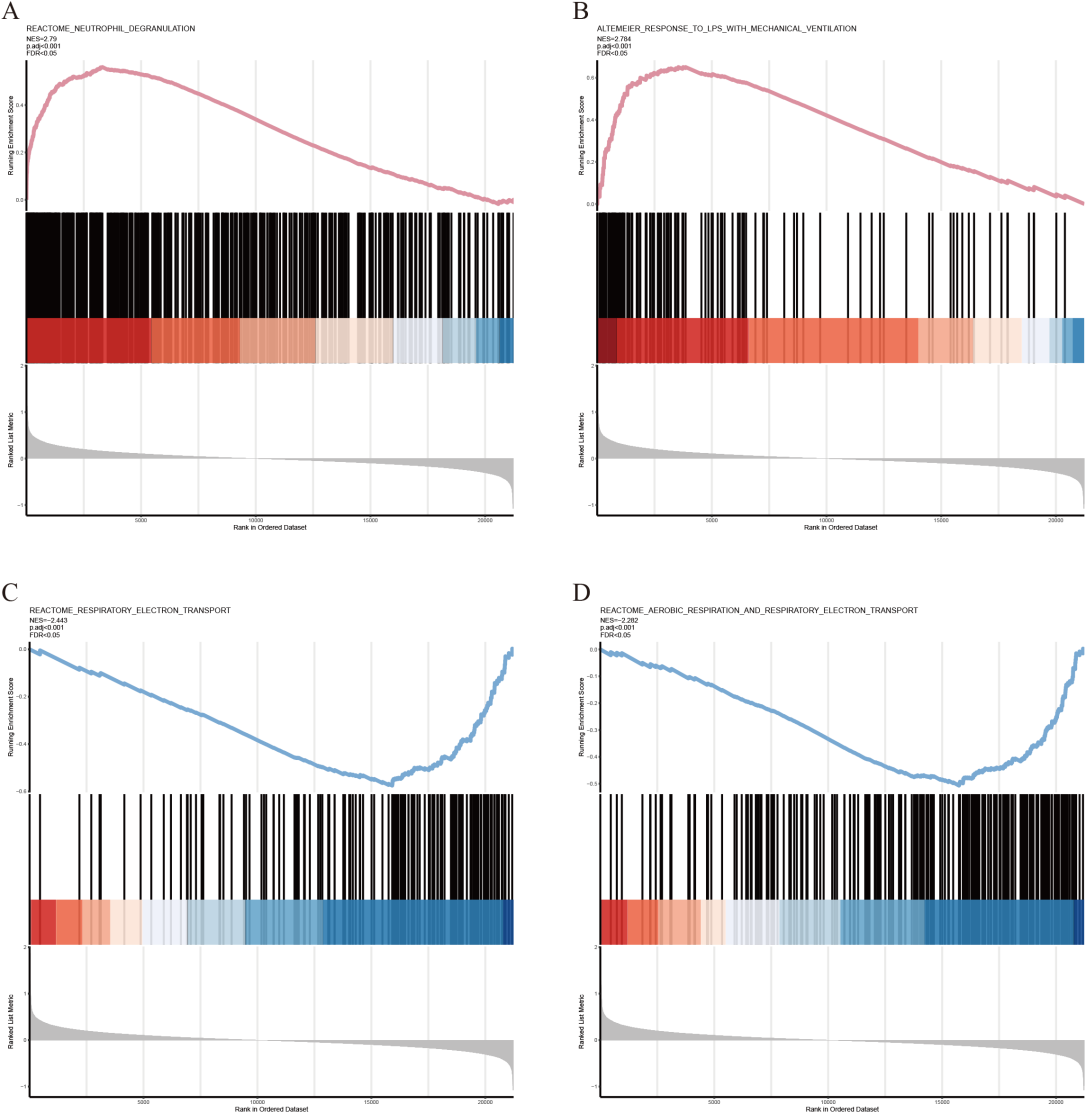
GSEA partial results. **(A)** Neutrophil degranulation pathway. **(B)** Response to LPS with mechanical ventilation pathway. **(C)** Respiratory electron transport chain pathway. **(D)** Aerobic respiration and respiratory electron transport chain pathways.

KEGG and GO analyses were performed on the identified AIDEGs. The results revealed that the AIDEGs were associated with 97 KEGG pathways (**Supplementary Data S5**), with key pathways such as MAPK, PI3K-Akt, osteoclast differentiation, apoptosis, necroptosis, and IL-17 signaling identified as potentially involved in the progression of IVDD (**Figure 2A**). The top 10 enriched GO terms (**Supplementary Data S4**) were visualized using circos plots (**Figures 2B–D**), indicating that aging-related IVDD genes are primarily associated with immune response, stress response, protein and cytokine binding, vesicle-mediated cellular transport, and secretion processes. Separate enrichment analyses were conducted for upregulated and downregulated AIDEGs, with the results presented in **Figures 2E, F**. GSEA results (**Supplementary Data S8**) are shown in **Figures 3A–D**, demonstrating activation of pathways such as neutrophil degranulation, and response to LPS with mechanical ventilation during the aging-related progression of IVDD. Pathways like aerobic respiration and electron transport chain were suppressed. In summary, the enrichment analysis underscores the critical roles of AIDEGs in multiple key biological pathways.

### 3.3 WGCNA results

**Figure 4:**
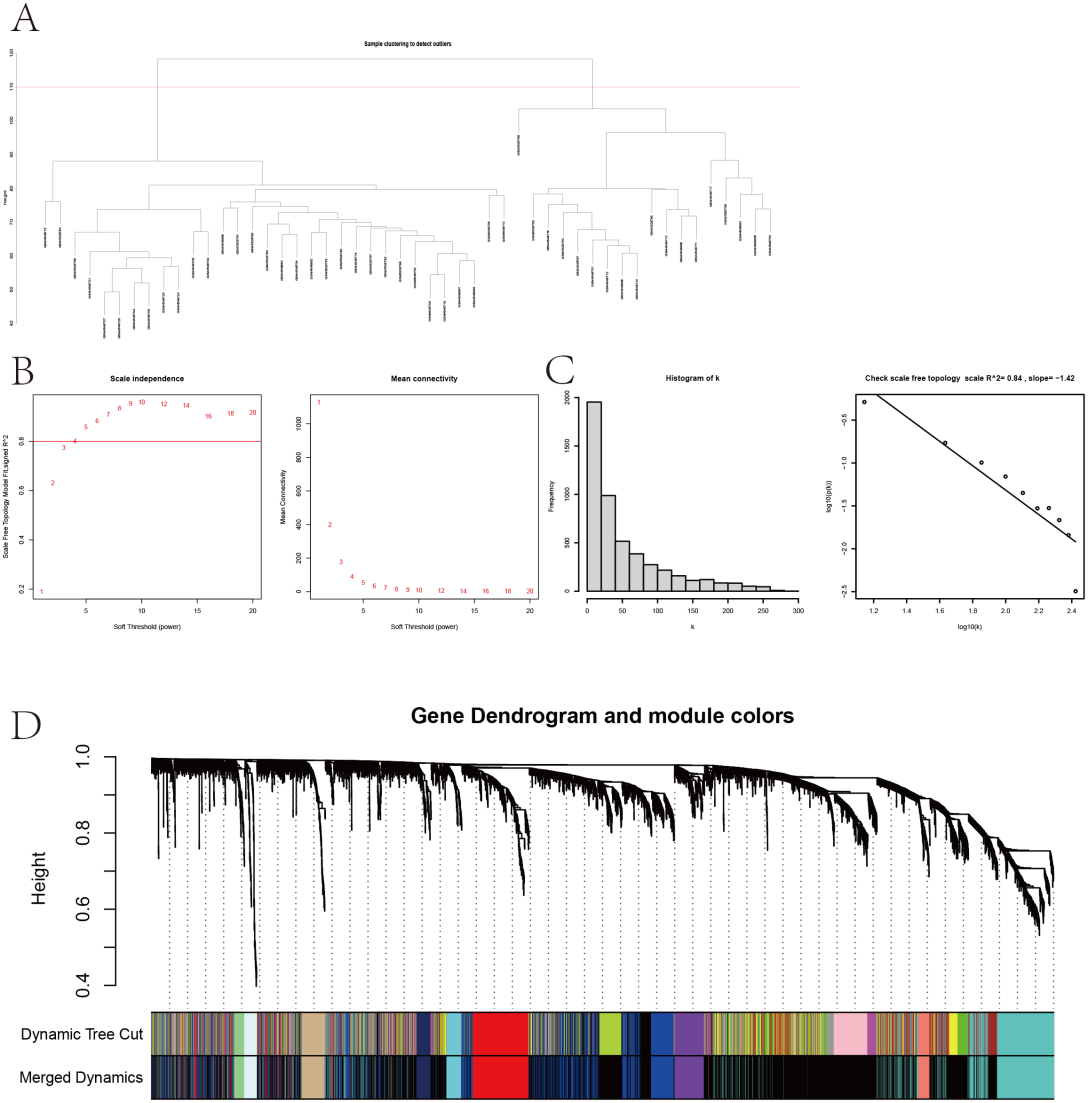
Sample clustering and WGCNA. **(A)** Sample clustering dendrogram with a cut height of 110. **(B)** Soft-thresholding power selection. **(C)** Verification of the scale-free network. **(D)** Merged dynamic tree cut after module merging.

**Figure 5:**
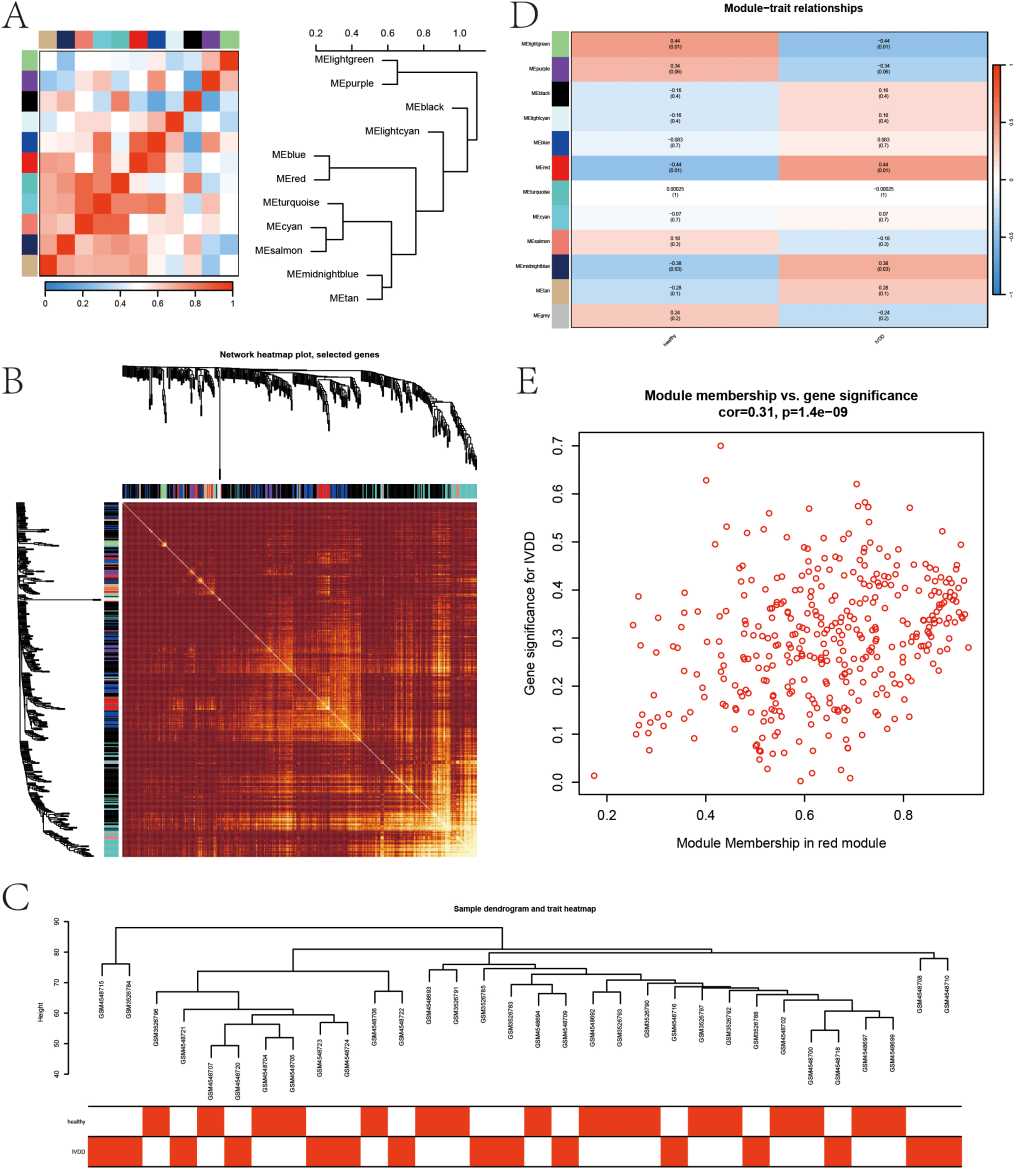
Gene network visualization and module-trait relationships in WGCNA. **(A)** Module eigengene network. **(B)** Gene network heatmap. **(C)** Sample clustering dendrogram combined with a trait heatmap. **(D)** Heatmap of module-trait relationships. **(E)** Scatter plot showing the correlation between genes in the red module and IVDD.

The sample clustering dendrogram (**Figure 4A**) identified 18 outliers removed at a cut height of 110, leaving 32 samples for analysis. The soft-thresholding power was determined as 5 (**Figure 4B**), and the network conformed to a scale-free topology (**Figure 4C**). Modules with similarity over 0.75 were merged into 12 modules, as shown in the merged dynamic tree cut diagram (**Figure 4D**). The module eigengene network (**Figure 5A**) shows correlations among eigengenes, with red indicating positive and blue negative correlations. The gene network heatmap (**Figure 5B**) illustrates co-expression among 400 genes, with brighter colors indicating stronger relationships. Sample clustering with trait heatmaps (**Figure 5C**) distinguishes healthy from diseased samples. Finally, the module-trait relationship heatmap (**Figure 5D**) shows a significant link between the red module and IVDD (*P* = 0.0107), visualized further in a scatter plot (**Figure 5E**), with 365 genes selected for further analysis.

### 3.4 Core gene selection and immune infiltration analysis

**Figure 6:**
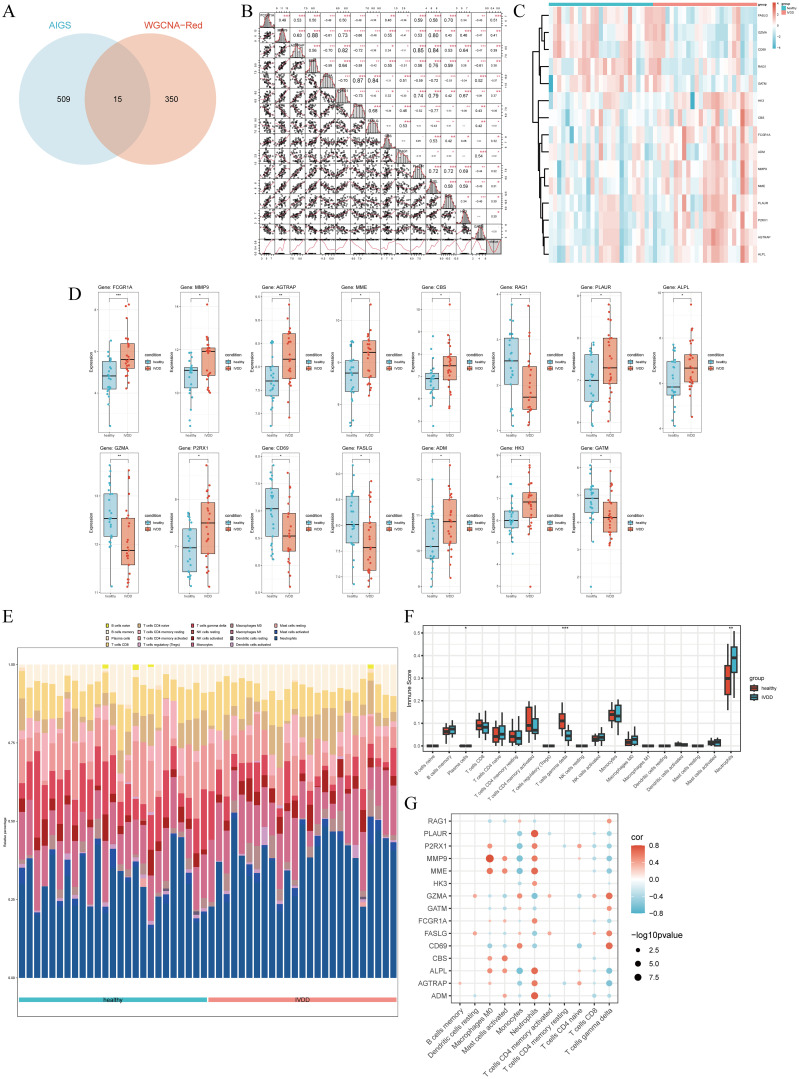
Correlation between hub genes and immune infiltration patterns. **(A)** Venn diagram showing the intersection of AIDEGs with WGCNA module genes. **(B)** Correlation analysis among hub genes. **(C)** Heatmap of hub gene expression. **(D)** Differential expression of hub genes between the healthy and IVDD groups. **(E)** Immune cell infiltration in the healthy and IVDD groups. **(F)** Expression differences of various immune cells between the healthy and IVDD groups. **(G)** Correlation between hub genes and immune cells. *p < 0.05, **p < 0.01, ***p < 0.001.

The intersection of AIDEGs with WGCNA module genes identified 15 hub genes (**Figure 6A**), with *MMP9* and *MME* showing the highest correlation, followed by *GZMA-CD69* and *AGTRAP-PLAUR* (**Figure 6B**). The PPI network reveals a highly interconnected structure comprising 31 egdes with numerous interactions (**Supplementary Figure S1**). Network analysis identified several hub genes, such as FCGR1A, CD69, FASLG, MMP9, GZMA, and CBS, which exhibit high connectivity and occupy central positions within their respective network clusters. These findings suggest that these genes play crucial regulatory roles in the biological processes under investigation. **Figures 6C, D** illustrate the expression levels of hub genes in the original dataset and their differential expression between the healthy and IVDD groups. *FCGR1A* exhibited the most significant difference (*P* < 0.001), and all other genes showed significant differences between the two groups (*P* < 0.05) (**Supplementary Table S1**). Immune cell infiltration analysis revealed that neutrophils, monocytes, and activated CD4+ T cells were the most highly expressed cell types (**Figure 6E**). Among them, neutrophil expression was significantly elevated in the IVDD group, while γδ T cells were significantly downregulated (**Figure 6F**). **Figure 6G** shows correlations between hub genes and immune cells, with red for positive and blue for negative correlations.

### 3.5 Construction and validation of the predictive model

Following random allocation, the training set comprised 36 samples, while the validation set included 14 samples. Comparative analysis between the two datasets, as presented in **Supplementary Table S2**, revealed no significant differences across all genes (*P* > 0.05), demonstrating the comparability of the training and validation sets.

### 3.6 Logistic regression

**Figure 7:**
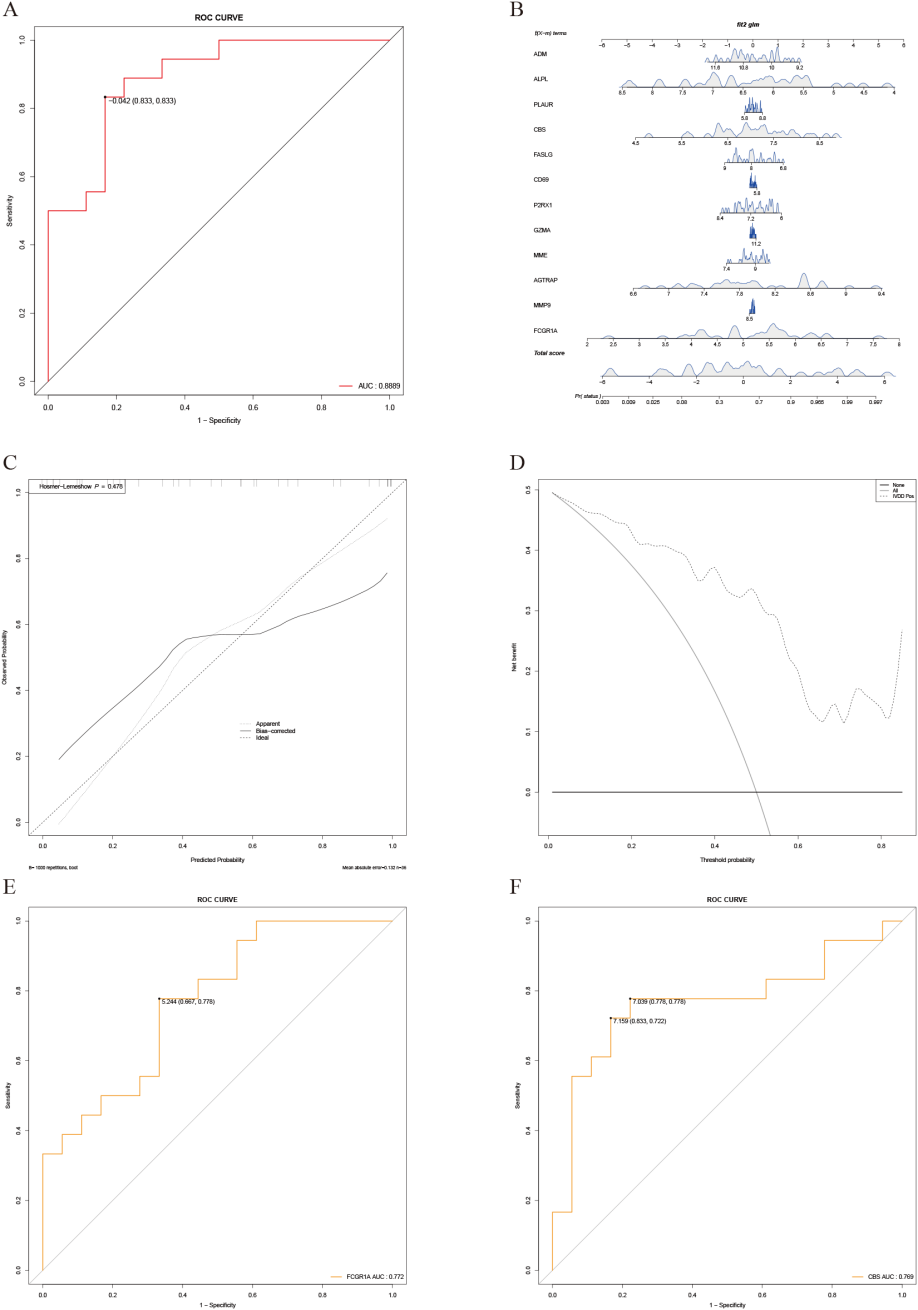
Construction and validation of the logistic regression prediction model. **(A)** ROC curve of the multivariate logistic regression prediction model, with an AUC of 0.91. **(B)** Nomogram generated based on the logistic regression prediction model. **(C)** Calibration curve showing good performance of the model on the training set. However, bias-corrected results indicate some calibration deviations in the mid-to-high probability range. The HL test (P=0.4783) suggests no significant calibration issues. **(D)** Decision curve analysis (DCA). The x-axis represents the predicted probability threshold, and the y-axis indicates net benefit, demonstrating the clinical utility of the model across varying thresholds. **(E)** ROC curve for univariate analysis of *FCGR1A*, showing a predictive performance with an AUC of 0.772. **(F)** ROC curve for univariate analysis of *CBS*, showing a predictive performance with an AUC of 0.796.

Univariate logistic regression analysis was performed for all hub genes, with results shown in **Supplementary Table S3**. Genes with *P* < 0.2 were included in multivariate logistic regression, and stepwise regression identified *FCGR1A* and *CBS* as key variables. *FCGR1A* remained significantly associated with IVDD after adjustment, suggesting its role as a key aging-related gene in IVDD. Collinearity diagnostics showed no multicollinearity, with variance inflation factors for *FCGR1A* and *CBS* being 1.002392. The multivariate logistic regression model demonstrated good predictive performance, with a ROC curve AUC of 0.889 (**Figure 7A**). A nomogram was constructed to visually present individualized risk predictions (**Figure 7B**). The C-index was 0.89 (95% CI: 0.79–0.99), and the Hosmer–Lemeshow test (*P* = 0.478) confirmed good calibration, supported by a calibration curve where the “Apparent” line aligned closely with the “Ideal” line. However, slight deviations in the “Bias-corrected” line suggested potential overfitting and calibration limitations in higher probability ranges (**Figure 7C**). DCA showed the model provided a net positive benefit for IVDD intervention (**Figure 7D**). Stepwise regression confirmed *FCGR1A* and *CBS* as significant predictors, with univariate ROC AUC values of 0.772 and 0.769, respectively, indicating moderate predictive accuracy (**Figures 7E, F**) (13).

### 3.7 RF

**Figure 8:**
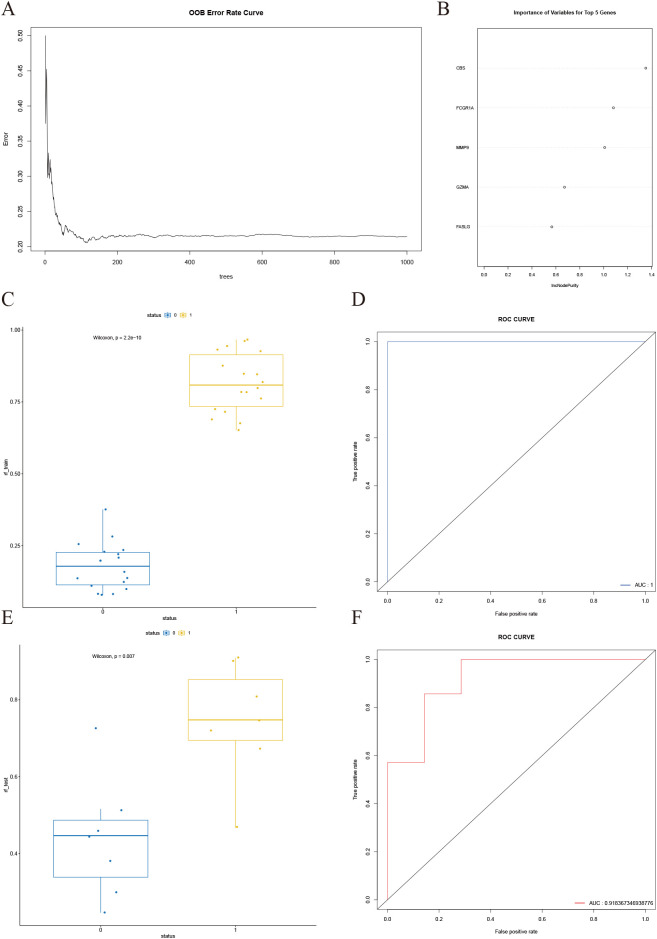
Construction and validation of the random forest model. **(A)** OOB error curve, showing that the overall error rate stabilizes when the number of trees exceeds approximately 200. **(B)** Variable importance plot, highlighting the top five most important genes ranked by their contribution to model performance. **(C)** Box plot of predicted values for different status groups in the training set, with Wilcoxon test p-value < 0.001. **(D)** ROC curve for the training set, demonstrating an AUC of 1. **(E)** Box plot of predicted values for different status groups in the test set, with Wilcoxon test p-value < 0.05. **(F)** ROC curve for the test set, showing an AUC of 0.91.

The OOB error curve is presented in **Figure 8A**. As the number of trees increases, the error rate initially fluctuates but stabilizes when the number of trees exceeds approximately 200. Subsequently, gene importance was assessed (**Figure 8B**). The x-axis represents the increase in node purity, an indicator of each variable’s contribution to classification accuracy. Higher values signify greater predictive importance. The top five ranked genes identified were *FCGR1A*, *CBS*, *GZMA*, *MMP9*, and *FASLG*. The constructed RF model was validated using both the training set (**Figures 8C, D**) and the test set (**Figures 8E, F**). Results indicate that the RF model achieves robust predictive performance in both internal and external validations, with an accuracy of 0.7857, a sensitivity of 0.7143, and a specificity of 0.5871 on the validation set.

### 3.8 Support vector machine

**Figure 9:**
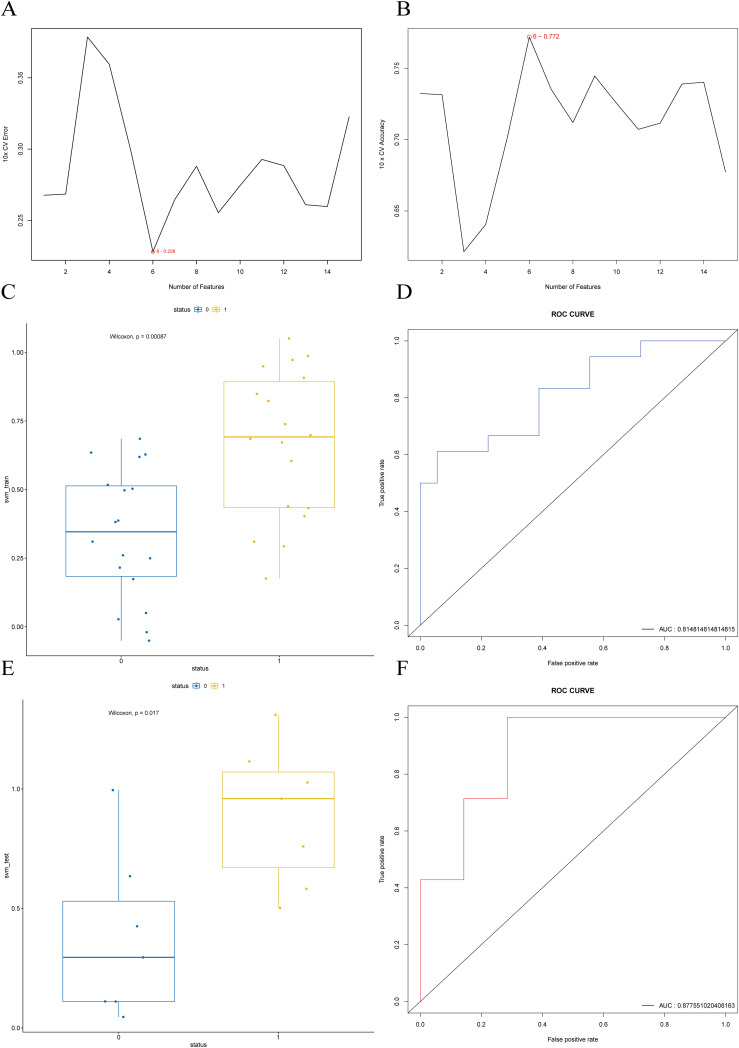
SVM-RFE feature selection and SVM model construction and validation. **(A)** Feature selection cross-validation error based on the training set. **(B)** Feature selection cross-validation accuracy based on the training set. **(C)** Boxplot of predicted values distribution for each status group in the training set, with p-value < 0.001. **(D)** ROC curve for the training set, with AUC of 0.87. **(E)** Boxplot of predicted values distribution for each status group in the test set, with p-value < 0.05. **(F)** ROC curve for the test set, with AUC of 0.86.

The results of feature selection using SVM-RFE are shown in **Figures 9A, B**, where the model achieves the lowest error rate with six features. Based on the feature importance ranking (**Supplementary Table S4**), the top six features were selected and used for the subsequent SVM model construction. The performance of the resulting SVM model was validated using both the training set (**Figures 9C, D**) and the test set (**Figures 9E, F**). The results demonstrate that the constructed SVM model exhibits strong predictive performance both internally and externally, with an accuracy of 0.8571, a sensitivity of 0.7143, and a specificity of 1 on the validation set.

### 3.9 LASSO regression

**Figure 10:**
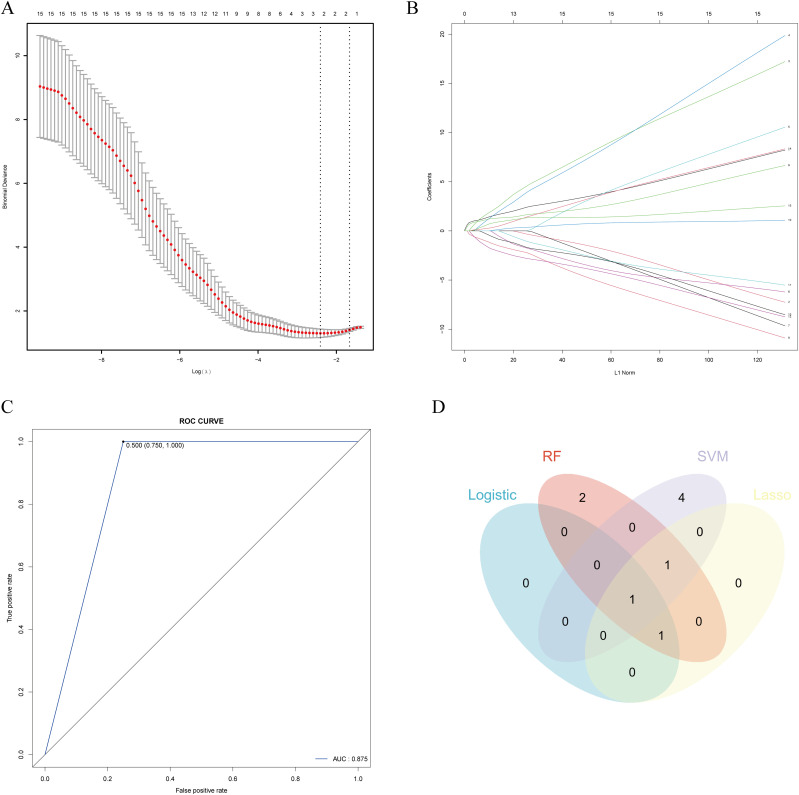
Lasso regression, K-NN and integration of machine learning results. **(A)** Ten-fold cross-validation curve of λ in the LASSO regression model. **(B)** Coefficient path diagram of the LASSO regression model, illustrating that as the L1 norm increases, more features are incorporated into the model. **(C)** ROC curve of the K-NN model, with an AUC of 0.875. **(D)** Integration of feature genes identified through multiple machine learning approaches.

The 10-fold cross-validation plot for λ in the LASSO regression model is shown in **Figure 10A**. The λ value that minimizes the deviation is 0.08202, retaining three features: *FCGR1A*, *FASLG*, and *CBS*. The coefficient path plot (**Figure 10B**) illustrates the relationship between λ and feature coefficients. The x-axis represents the L1 norm (the scale of the regularization path), showing how each feature coefficient changes with varying λ. The y-axis indicates the magnitude of the coefficients. As the L1 norm increases from left to right (corresponding to a decreasing λ and weaker penalty), more features are incorporated into the model, and their coefficient magnitudes progressively increase, reflecting their growing influence on the model.

### 3.10 KNN

The KNN algorithm (K = 2) was used to classify and predict the validation set samples by calculating the class labels of the two nearest neighbors for each sample. To assess the stability and reliability of the model, we performed five-fold cross-validation on the training set. The results thereof showed that the overall accuracy of the KNN model on the validation set was 0.875 (**Figure 10C**), indicating that the model has high accuracy in classification tasks. The confusion matrix results showed a sensitivity of 0.750 and specificity of 1.000, demonstrating that the KNN model constructed using hub genes has high accuracy in sample classification.

### 3.11 Integration of machine learning results

The intersection of features identified by logistic regression, RF, SVM, and LASSO revealed *FCGR1A* as a common feature across all methods, while *CBS* and *FASLG* were identified by three methods each (**Figure 10D**). These three key genes were selected for further experimental validation.

### 3.12 ELISA

**Figure 11:**
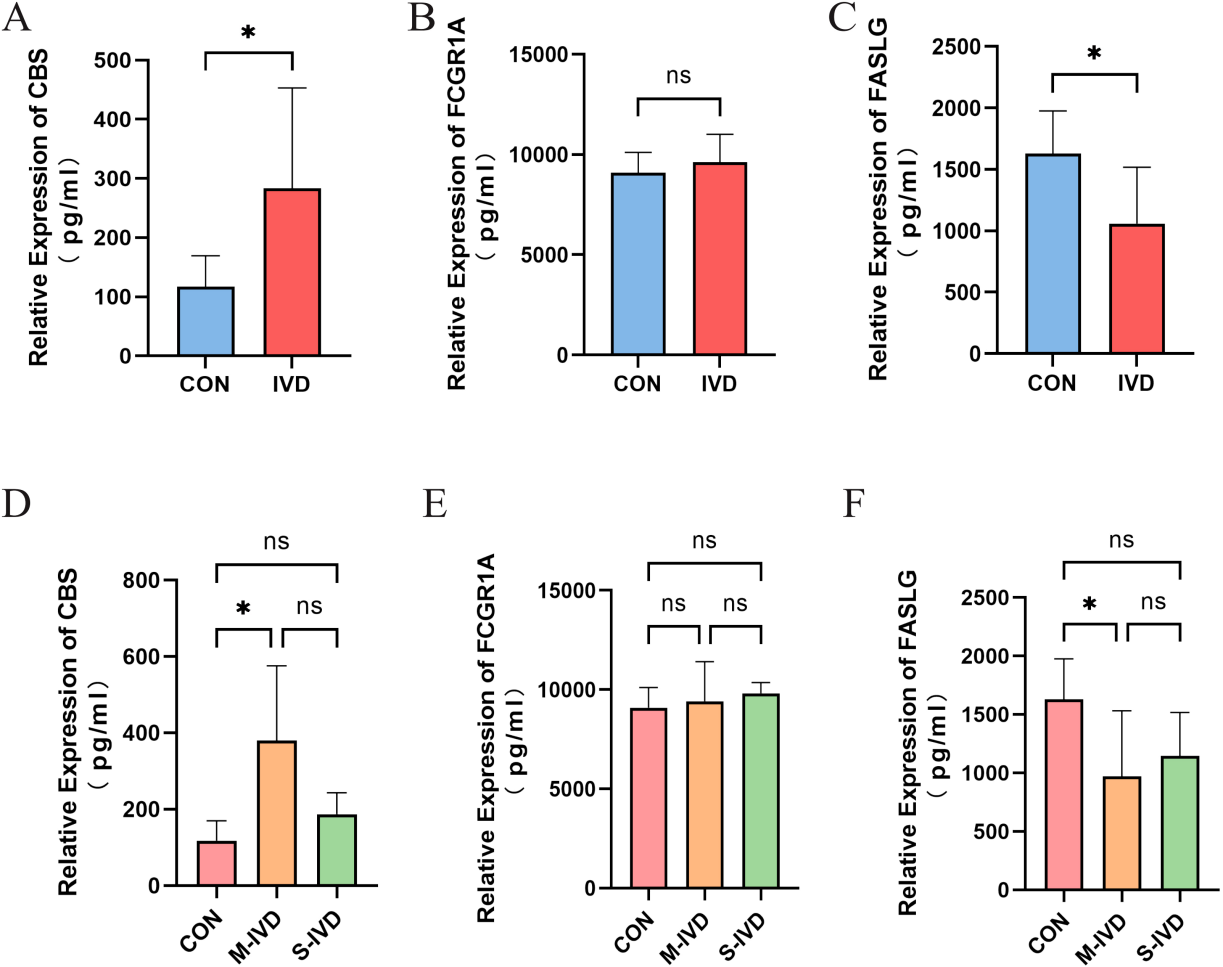
ELISA results of key genes. **(A-C)** Comparisons of the expression of three key genes between the control group and the IVDD group. **(D-F)** Expression of three key genes among IVDD patients with different Pfirrmann degeneration grades. *P<0.05. ns, not statistically significant (p ≥ 0.05).

The demographic and clinical information of all patients included in the study is presented in **Supplementary Table S5**. A total of 17 patients were enrolled, with patients with IVDD further stratified into subgroups based on the Pfirrmann grading system: the control group (healthy) comprised 7 individuals, the moderate IVDD group included 5 patients, and the severe IVDD group consisted of 5 patients. The Pfirrmann grading was independently assessed by at least two spine surgeons to ensure consistency and reliability in the classification process. The representative MRI images of typical subjects from each group are presented in **Supplementary Figure S2**. ELISA results are shown in **Supplementary Table S6**. First, we compared the healthy control group with all patients with IVDD. The results demonstrated that *CBS* expression was significantly upregulated in these patients (*P* = 0.0123), while *FASLG* expression was significantly downregulated (*P* = 0.0185). No significant difference was observed in *FCGR1A* expression between the two groups (*P* = 0.4747) (**Figures 11A–C**).

Subsequently, the patients with IVDD were divided into moderately degenerated (M-IVD) and severely degenerated (S-IVD) groups, and an analysis of variance (ANOVA) with *post hoc* pairwise comparisons was performed. The results revealed significant differences in the expression levels of *CBS* (*P* = 0.0365) and *FASLG* (*P* = 0.0437) among the three groups, while *FCGR1A* showed no significant differences (*P* = 0.6311). Specifically, *CBS* expression was significantly upregulated (*P* = 0.0380) and *FASLG* expression significantly downregulated (*P* = 0.0470) in the M-IVD group compared to the control group. However, no significant changes in the expression of the three key genes were observed in the S-IVD group.

To explore the relationship between age and the expression of these key genes, a linear regression analysis was conducted, with age included as a variable. The results, presented in **Supplementary Table S7**, showed significant differences in *CBS* and *FASLG* expression levels under different subgroup conditions. Compared to the control group, *CBS* expression was significantly elevated in the M-IVD group (*β* = 249.8, *P* = 0.0021), while *FASLG* expression was significantly reduced (*β* = -664.2, *P* = 0.0241). When age was included as a covariate, it exerted a slight downregulatory effect on *CBS* and *FASLG* expression (*β_CBS_* = -5.016, *β_FASLG_* = -2.046), while *FCGR1A* expression exhibited a weak upward trend with age (*β_FCGR1A_* = 1.959). However, these effects were not statistically significant (*P_CBS_* = 0.1838, *P_FASLG_* = 0.8881, *P_FCGR1A_* = 0.9645), indicating that the variations in key gene expression were primarily driven by degeneration grade, with age playing a minor regulatory role (**Figures 11D–F**).

In summary, *CBS* and *FASLG* exhibit significant expression differences in the blood of patients with IVDD, particularly in those with moderate degeneration, while no significant changes were observed in patients with severe degeneration. The role of *FCGR1A* in the pathological process of IVDD requires further investigation.

## 4 Discussion

As the population ages, more elderly patients suffer from LBP due to IVDD, posing substantial physical, psychological, and economic burdens (3). Consequently, an exhaustive exploration of the molecular biological mechanisms that underpin IVDD is essential to enhance existing clinical diagnostic and therapeutic strategies. Many studies have shown that the onset and advancement of IVDD are intricately linked to factors including inflammatory responses, immune modulation, cellular aging, and metabolic dysregulation (4, 14). However, at present, no research has pinpointed early aging-related biomarkers of IVDD in complete blood samples. Consequently, this study utilizes bioinformatics analysis to uncover AIDEGs in the whole blood of patients with IVDD. Machine learning algorithms were employed to develop diagnostic models that evaluate the predictive capability of the chosen AIDEGs and further identify crucial genes. The objective was to furnish novel targets for the diagnosis and treatment of IVDD.

By analyzing two IVDD whole blood samples from the GSE datasets, we identified a total of 3,813 DEGs. We then integrated ARGs from multiple databases, and the intersection of these datasets yielded 524 AIDEGs. Enrichment analysis revealed that these genes are primarily involved in apoptosis, inflammatory response, immune response, and oxidative stress. Mitochondria are the primary sites for aerobic respiration in most eukaryotic cells, and mitochondrial dysfunction and aging are bidirectionally related and mutually reinforcing (15). In skeletal muscle, the phosphorylation of mitochondrial respiration declines with aging (16). In this study, the GSEA results suggest that aerobic respiration and electron transport processes may be inhibited during the aging-related development of IVDD. However, we cannot rule out the possibility that this result is related to the structural characteristics of the intervertebral disc itself, as the disc lacks blood vessels, and oxygen supply is achieved through diffusion via the endplate, because intervertebral disc cells tend to rely on anaerobic metabolism (17).

IVDD is closely associated with various immune cells, including macrophages, B cells, and T cells. Immune infiltration analysis revealed that neutrophils were significantly elevated in the whole blood samples of patients with IVDD, consistent with previous studies (18, 19). Neutrophils, as a critical component of innate immunity, are among the first white blood cell populations recruited to sites of inflammation in human blood. They play a pivotal role in the initiation and progression of inflammatory responses. Additionally, neutrophils contribute to tissue repair by clearing cellular debris, promoting angiogenesis (20), and inducing anti-inflammatory responses (21). Neutrophils may contribute to the pathogenesis and progression of IVDD through multiple mechanisms. Neovascularization has been identified as a critical factor in the onset and exacerbation of IVDD (22). Neutrophils are a significant source of vascular endothelial growth factor (VEGF) (23), as evidenced by impaired neovascularization observed in neutrophil-depleted mouse models of muscle injury (20).

Extracellular matrix (ECM) degradation is another key contributor to the progression of IVDD and is closely associated with various matrix metalloproteinases (MMPs) (24). Neutrophils are the sole cellular source of MMP-9, which induces disc degeneration by promoting the loss of type II collagen (25). Conversely, modic changes are highly associated with IVDD (26) and represent a significant specific feature in vertebral bodies of patients suffering from LBP (27). With the aging population, modic changes are becoming increasingly prevalent (28). The formation of neutrophil extracellular traps (NETs) may exacerbate modic changes by activating the complement system (29, 30), ultimately contributing to the progression of IVDD. Additionally, complement system activation is associated with angiogenesis and neurogenesis (31). Whether the ingrowth of nerves and blood vessels within the intervertebral disc is related to NET-mediated complement activation remains to be further investigated.

In various IVDD animal models, increased T-cell expression has been observed in NP tissues (32). γδT cells, a unique subset of regulatory T cells (Tregs), constitute a minor proportion of human peripheral blood. Based on the usage of their T-cell receptor variable (V) gene segments, they can be categorized into Vδ1 and Vδ2 T cells. These T-cell subsets are highly enriched in peripheral organs such as the skin, intestine, and lungs (33). They exert anti-inflammatory effects by secreting IL-10, IL-4, and TGF-β, expressing membrane-bound TGF-β complexes, or indirectly attracting myeloid-derived suppressor cells through IL-17 (34). Aging can lead to a reduction in naïve Tregs and an accumulation of memory Tregs, resulting in decreased T-cell immune diversity. This age-related imbalance in T-cell homeostasis is strongly associated with tumors, chronic inflammation, and autoimmune diseases (35). The role of γδT cells in the aging process remains unclear, and no studies have yet explored the relationship between this subset of T cells and IVDD. Our study found that γδT cells were downregulated in the peripheral blood of patients with IVDD. In skeletal system disorders, γδT cells—primarily Vγ6+ γδT cells—produce IL-17, which stimulates the proliferation of mesenchymal progenitor cells and the differentiation of osteoblasts, ultimately promoting fracture healing (36). Our findings revealed a significant reduction in γδT cells in the blood samples of patients with IVDD, while their expression was markedly elevated in degenerated NP tissue compared to normal intervertebral discs (4). This discrepancy may be attributed to the chemotactic recruitment of γδT cells to the degenerated disc or could result from individual differences among samples or variations in detection methods (37).

Subsequently, we identified three key genes, *FCGR1A*, *CBS* and *FASLG*, using multiple machine learning algorithms. The *FCGR1A* gene encodes the functional high-affinity IgG Fc receptor CD64 (or FcγR1A), which mediates various immune functions (38). Under normal conditions, CD64 is primarily expressed on monocytes and macrophages. During infection or inflammation, CD64 expression increases significantly, particularly on the surface of neutrophils, where its levels can rapidly escalate within a short period (39). In transgenic mice, CD64-induced allergic reactions are predominantly mediated by neutrophils (40). In addition, M1 macrophage polarization is one of the key factors in the pathogenesis of IVDD, leading to the release of various inflammatory mediators (5). CD64 is significantly upregulated in M1 macrophages, while it is typically downregulated in M2 macrophages. This expression difference makes CD64 a distinctive marker for identifying M1 pro-inflammatory macrophages and suggests its potential clinical application in regulating chronic inflammation resulting from M1 macrophage dysregulation (41, 42). In the synovium of patients with rheumatoid arthritis, *FCGR1A* expression is upregulated and positively correlated with the expression of various matrix metalloproteinases (43), a trend consistent with changes observed in degenerated intervertebral discs. In summary, FCGR1A may promote the progression of IVDD through multiple pathways. However, current related research is limited, and factors such as detection methods and sample collection may influence the final experimental validation. Further experimental studies are needed in the future.

*CBS* is the first rate-limiting enzyme in the transsulfuration pathway. It catalyzes the condensation of cysteine and homocysteine to produce hydrogen sulfide (H_2_S), which mediates various physiological and pathological processes in the body (44). *CBS* deficiency has been linked to various diseases, including osteoporosis, lens dislocation, and thrombosis (45). Research by Saha et al. revealed that silencing *CBS*, resulting in reduced H_2_S expression, inhibits Sp1-mediated expression of vascular endothelial growth factor receptor 2 (VEGFR-2) and neuropilin-1 (NRP-1), ultimately leading to endothelial dysfunction (46). Song et al. identified a critical role for Sp1 in IVDD through bioinformatics and experimental approaches (47). Sp1, as a trans-activator, mediates the growth factor-regulated expression of CBS. Moreover, in the GO analysis presented in our study, both Sp1 and CBS were found to be involved in the hydrogen sulfide biosynthetic process, suggesting that the Sp1/CBS pathway may play an important role in the development of IVDD. Meanwhile, H_2_S is crucial for protecting cells against apoptosis, mitochondrial damage, and endoplasmic reticulum (ER) stress, which further underscores the significant role of *CBS* in numerous physiological processes. Alterations in *CBS* and H_2_S activity are associated with IVDD. Notably, in herniated disc tissues, especially in cases of ruptured disc extrusion, *CBS* and H_2_S expression are significantly upregulated (48). On one hand, H_2_S may alleviate disc degeneration by improving mitochondrial function, inhibiting ER stress, and activating protective cellular signaling pathways (49). On the other hand, the high rate of H_2_S generation could promote NP cell apoptosis, contributing to the progression of degeneration (48). In terms of immunity, H_2_S demonstrates anti-neutrophil properties (50). However, high concentrations and rapid release of H_2_S can promote neutrophil apoptosis and inhibit chemotactic responses (51–53). Furthermore, Tregs express higher levels of *CBS* compared to other CD4^+^ T-cell subsets (54). In this study, we observed an upregulation of *CBS* expression in the whole blood samples of patients with IVDD. *CBS* expression peaked in M-IVDD patients but declined in S-IVDD patients, aligning with the findings of Xu et al. in intervertebral disc tissues (49). This suggests that the anti-inflammatory activity of H_2_S mediated by *CBS* may gradually diminish as IVDD progresses. Overall, *CBS* may facilitate the advancement of IVDD by reducing inflammation and engaging in angiogenesis, although this is predominantly driven by H_2_S; however, this necessitates additional experimental confirmation.

*Fas ligand (FASLG*), or *FasL*, is a type II transmembrane protein belonging to the tumor necrosis factor superfamily. It is primarily expressed by T cells and other lymphocytes, and induces apoptosis by binding to the *Fas* receptor, thereby helping to regulate T-cell numbers and maintain immune system balance (55). The *Fas* ligand system plays a crucial role in maintaining the immune privilege of the intervertebral disc. During the progression of IVDD, many studies have reported that *FASLG* expression is predominantly upregulated, promoting IVDD by inducing apoptosis of NP cells via the *Fas* system. However, there are also studies reporting decreased *FASLG* expression and functional impairment in IVDD (56). For example, Liu et al. observed that the expression of *Fas* in degenerative NP cells was significantly lower than that in normal NP cells, which compromised their immune privilege. Moreover, the upregulation of *Fas* in normal NP cells could induce the apoptosis of co-cultured macrophages and CD8+ T cells (57). This imbalance interaction between NP cells and immune cells mediated by *Fas* would trigger an enhanced immune response and aggravate IVDD. In our study, a significant down-regulation of *FASLG* expression was observed in the whole blood samples of M-IVDD patients, potentially indicating activation of *FASLG*-related immune mechanisms in these individuals.

Due to the unique structural characteristics of intervertebral discs, gene expression levels may vary between different sample sources, such as blood and NP. To address this issue, our study utilized datasets with consistent sample origins, ensuring that the results were not influenced by discrepancies in sample sources. Finally, we validated the expression of three key genes in the peripheral blood of IVDD patients using ELISA. The results confirmed that *CBS* and *FCGR1A* exhibit significant expression changes in cases of moderate degeneration, while these changes gradually diminish as degeneration progresses further. The underlying mechanisms require further investigation.

Based on whole blood samples from patients with IVDD, we identified AIDEGs as key contributors to the initiation and progression of IVDD through various biological pathways. Through the application of multiple machine learning algorithms, FCGR1A, CBS, and FASLG were identified as three key genes, and *in vitro* ELISA experiments validated that CBS and FASLG exhibit significant differential expression in the peripheral blood of patients with moderate IVDD. These findings provide strong evidence for the potential use of CBS and FASLG as non-invasive biomarkers for IVDD diagnosis and prognosis.

Beyond biomarker identification, this study highlights the utility of integrating bioinformatics and machine learning to uncover molecular mechanisms and diagnostic targets in complex, multifactorial diseases like IVDD. By focusing on blood-based biomarkers, our research addresses the critical need for accessible, minimally invasive diagnostic tools, which could facilitate early intervention and improve patient outcomes.

However, this study has some limitations. First, due to the unique structural characteristics of the intervertebral disc, the sensitivity and specificity of blood samples for the early detection of IVDD require further experimental validation. Moreover, the datasets used lack detailed clinical information, such as the specific ages of participants and the severity of disc degeneration. In addition, the clinical samples included in this study predominantly consist of young and middle-aged individuals. The integration of transcriptomic data from elderly populations could enhance the precision and generalizability of the findings. Consequently, age matching should be rigorously implemented in future research to minimize confounding effects and improve the robustness of the results. Future studies should validate these findings in larger, more diverse cohorts to reduce the potential for false positives, using rigorous statistical methods and independent validation. Investigating the longitudinal relationship between biomarker expression and IVDD progression, along with exploring the molecular mechanisms of CBS and FASLG in IVDD pathogenesis, will be essential to confirm their therapeutic potential.

## 5 Conclusion

This study not only advances our understanding of the molecular biology of IVDD, but also lays the foundation for the development of blood-based diagnostic tools and targeted therapeutic strategies. Such advancements could significantly reduce the burden of IVDD by enabling earlier diagnosis and more personalized treatment options.

## Data Availability

The original contributions presented in the study are included in the article/**Supplementary Material**. Further inquiries can be directed to the corresponding authors.

## Supplementary material

Supplementary Figure 1 PPI network of 15 hub genes. Each node represents a gene, with interactions between them indicated by connecting edges. Nodes are colored based on functional clusters. The interactions are visualized using various edge colors to highlight different types of relationships.

Supplementary Figure 2 Typical MRI findings for different groups: left to right—T2, T1 sagittal, and axial images. **(A–C)** CON-1, male, 27, Pfirrmann grade 1. **(D–F)** M-IVDD-2, male, 28, Pfirrmann grade 3. **(G–I)** S-IVDD-5, female, 40, Pfirrmann grade 4.

## References

[1] Knezevic NN Candido KD Vlaeyen JWS Van Zundert J Cohen SP. Low back pain. Lancet (London England). (2021) 398:78–92. doi: 10.1016/s0140-6736(21)00733-9 34115979

[2] López-Otín C Blasco MA Partridge L Serrano M Kroemer G. Hallmarks of aging: An expanding universe. Cell. (2023) 186:243–78. doi: 10.1016/j.cell.2022.11.001 36599349

[3] Wong CK Mak RY Kwok TS Tsang JS Leung MY Funabashi M. Prevalence, incidence, and factors associated with non-specific chronic low back pain in community-dwelling older adults aged 60 years and older: A systematic review and meta-analysis. J pain. (2022) 23:509–34. doi: 10.1016/j.jpain.2021.07.012 34450274

[4] Wang M Wang H Wang X Shen Y Zhou D Jiang Y. Identification of cellular senescence-related genes and immune cell infiltration characteristics in intervertebral disc degeneration. Front Immunol. (2024) 15:1439976. doi: 10.3389/fimmu.2024.1439976 39328407 PMC11424418

[5] Feng P Che Y Gao C Zhu L Gao J Vo NV. Immune exposure: how macrophages interact with the nucleus pulposus. Front Immunol. (2023) 14:1155746. doi: 10.3389/fimmu.2023.1155746 37122738 PMC10140429

[6] Jiang X Wu J Guo C Song W. Key lncRNAs associated with oxidative stress were identified by GEO database data and whole blood analysis of intervertebral disc degeneration patients. Front Genet. (2022) 13:929843. doi: 10.3389/fgene.2022.929843 35937989 PMC9353269

[7] Hu X Ni S Zhao K Qian J Duan Y. Bioinformatics-led discovery of osteoarthritis biomarkers and inflammatory infiltrates. Front Immunol. (2022) 13:871008. doi: 10.3389/fimmu.2022.871008 35734177 PMC9207185

[8] Greener JG Kandathil SM Moffat L Jones DT. A guide to machine learning for biologists. Nat Rev Mol Cell Biol. (2022) 23:40–55. doi: 10.1038/s41580-021-00407-0 34518686

[9] Wang Y Dai G Jiang L Liao S Xia J. Microarray analysis reveals an inflammatory transcriptomic signature in peripheral blood for sciatica. BMC neurol. (2021) 21:50. doi: 10.1186/s12883-021-02078-y 33535986 PMC7856817

[10] Zhou J Huang J Li Z Song Q Yang Z Wang L. Identification of aging-related biomarkers and immune infiltration characteristics in osteoarthritis based on bioinformatics analysis and machine learning. Front Immunol. (2023) 14:1168780. doi: 10.3389/fimmu.2023.1168780 37503333 PMC10368975

[11] Huang K Gong H Guan J Zhang L Hu C Zhao W. AgeAnno: a knowledgebase of single-cell annotation of aging in human. Nucleic Acids Res. (2023) 51:D805–d15. doi: 10.1093/nar/gkac847 PMC9825500 36200838

[12] Shen W Song Z Zhong X Huang M Shen D Gao P. Sangerbox: A comprehensive, interaction-friendly clinical bioinformatics analysis platform. iMeta. (2022) 1:e36. doi: 10.1002/imt2.36 38868713 PMC10989974

[13] Mandrekar JN. Receiver operating characteristic curve in diagnostic test assessment. J Thorac Oncol. (2010) 5:1315–6. doi: 10.1097/JTO.0b013e3181ec173d 20736804

[14] Francisco V Pino J González-Gay M Lago F Karppinen J Tervonen O. A new immunometabolic perspective of intervertebral disc degeneration. Nat Rev Rheumatol. (2022) 18:47–60. doi: 10.1038/s41584-021-00713-z 34845360

[15] Wen P Zheng B Zhang B Ma T Hao L Zhang Y. The role of ageing and oxidative stress in intervertebral disc degeneration. Front Mol biosci. (2022) 9:1052878. doi: 10.3389/fmolb.2022.1052878 36419928 PMC9676652

[16] Jedlička J Tůma Z Razak K Kunc R Kala A Proskauer Pena S. Impact of aging on mitochondrial respiration in various organs. Physiol Res. (2022) 71:S227–s36. doi: 10.33549/physiolres.934995 PMC9906668 36647911

[17] Frapin L Clouet J Delplace V Fusellier M Guicheux J Le Visage C. Lessons learned from intervertebral disc pathophysiology to guide rational design of sequential delivery systems for therapeutic biological factors. Adv Drug delivery Rev. (2019) 149-150:49–71. doi: 10.1016/j.addr.2019.08.007 31445063

[18] Zhao W Wei J Ji X Jia E Li J Huo J. Machine learning algorithm predicts fibrosis-related blood diagnosis markers of intervertebral disc degeneration. BMC Med Genomics. (2023) 16:274. doi: 10.1186/s12920-023-01705-6 37915003 PMC10619283

[19] Li W Ding Z Zhang H Shi Q Wang D Zhang S. The roles of blood lipid-metabolism genes in immune infiltration could promote the development of IDD. Front Cell Dev Biol. (2022) 10:844395. doi: 10.3389/fcell.2022.844395 35223859 PMC8864150

[20] Graca FA Stephan A Minden-Birkenmaier BA Shirinifard A Wang YD Demontis F. Platelet-derived chemokines promote skeletal muscle regeneration by guiding neutrophil recruitment to injured muscles. Nat Commun. (2023) 14:2900. doi: 10.1038/s41467-023-38624-0 37217480 PMC10203137

[21] Castanheira FVS Kubes P. Neutrophils and NETs in modulating acute and chronic inflammation. Blood. (2019) 133:2178–85. doi: 10.1182/blood-2018-11-844530 30898862

[22] Rätsep T Minajeva A Asser T. Relationship between neovascularization and degenerative changes in herniated lumbar intervertebral discs. Eur Spine J. (2013) 22:2474–80. doi: 10.1007/s00586-013-2842-1 PMC3886507 23736847

[23] Wang J. Neutrophils in tissue injury and repair. Cell Tissue Res. (2018) 371:531–9. doi: 10.1007/s00441-017-2785-7 PMC5820392 29383445

[24] Molinos M Almeida CR Caldeira J Cunha C Gonçalves RM Barbosa MA. Inflammation in intervertebral disc degeneration and regeneration. J R Soc Interf. (2015) 12:20141191. doi: 10.1098/rsif.2014.1191 PMC4528607 26040602

[25] Xu YQ Zhang ZH Zheng YF Feng SQ. Dysregulated miR-133a mediates loss of type II collagen by directly targeting matrix metalloproteinase 9 (MMP9) in human intervertebral disc degeneration. Spine. (2016) 41:E717–e24. doi: 10.1097/brs.0000000000001375 26656045

[26] Lee SH Son DW Lee JS Sung SK Lee SW Song GS. Relationship between endplate defects, modic change, facet joint degeneration, and disc degeneration of cervical spine. Neurospine. (2020) 17:443–52. doi: 10.14245/ns.2040076.038 PMC7338942 32615702

[27] Dudli S Fields AJ Samartzis D Karppinen J Lotz JC. Pathobiology of modic changes. Eur Spine J. (2016) 25:3723–34. doi: 10.1007/s00586-016-4459-7 PMC5477843 26914098

[28] Applebaum A Nessim A Cho W. Modic change: an emerging complication in the aging population. Clin Spine surge. (2022) 35:12–7. doi: 10.1097/BSD.0000000000001168 33769981

[29] Heggli I Teixeira GQ Iatridis JC Neidlinger-Wilke C Dudli S. The role of the complement system in disc degeneration and Modic changes. JOR spine. (2024) 7:e1312. doi: 10.1002/jsp2.1312 38312949 PMC10835744

[30] Wang H Wang C Zhao MH Chen M. Neutrophil extracellular traps can activate alternative complement pathways. Clin Exp Immunol. (2015) 181:518–27. doi: 10.1111/cei.12654 PMC4557387 25963026

[31] Mapp PI Walsh DA. Mechanisms and targets of angiogenesis and nerve growth in osteoarthritis. Nat Rev Rheumatol. (2012) 8:390–8. doi: 10.1038/nrrheum.2012.80 22641138

[32] Ye F Lyu FJ Wang H Zheng Z. The involvement of immune system in intervertebral disc herniation and degeneration. JOR spine. (2022) 5:e1196. doi: 10.1002/jsp2.1196 35386754 PMC8966871

[33] Ribot JC Lopes N Silva-Santos B. γδ T cells in tissue physiology and surveillance. Nat Rev Immunol. (2021) 21:221–32. doi: 10.1038/s41577-020-00452-4 33057185

[34] Peters C Kabelitz D Wesch D. Regulatory functions of γδ T cells. Cell Mol Life sci: CMLS. (2018) 75:2125–35. doi: 10.1007/s00018-018-2788-x PMC11105251 29520421

[35] Rocamora-Reverte L Melzer FL Würzner R Weinberger B. The complex role of regulatory T cells in immunity and aging. Front Immunol. (2020) 11:616949. doi: 10.3389/fimmu.2020.616949 33584708 PMC7873351

[36] Ono T Okamoto K Nakashima T Nitta T Hori S Iwakura Y. IL-17-producing γδ T cells enhance bone regeneration. Nat Commun. (2016) 7:10928. doi: 10.1038/ncomms10928 26965320 PMC4792964

[37] Kim CF Moalem-Taylor G. Interleukin-17 contributes to neuroinflammation and neuropathic pain following peripheral nerve injury in mice. J pain. (2011) 12:370–83. doi: 10.1016/j.jpain.2010.08.003 20889388

[38] Wu J Li Y Rendahl A Bhargava M. Novel human FCGR1A variants affect CD64 functions and are risk factors for sarcoidosis. Front Immunol. (2022) 13:841099. doi: 10.3389/fimmu.2022.841099 35371020 PMC8968912

[39] Li S Huang X Chen Z Zhong H Peng Q Deng Y. Neutrophil CD64 expression as a biomarker in the early diagnosis of bacterial infection: a meta-analysis. Int J Infect Dis. (2013) 17:e12–23. doi: 10.1016/j.ijid.2012.07.017 22940278

[40] Mancardi DA Albanesi M Jönsson F Iannascoli B Van Rooijen N Kang X. The high-affinity human IgG receptor FcγRI (CD64) promotes IgG-mediated inflammation, anaphylaxis, and antitumor immunotherapy. Blood. (2013) 121:1563–73. doi: 10.1182/blood-2012-07-442541 23293080

[41] Jin Y Wang Y Lin X. Identification of key gene targets for periodontitis treatment by bioinformatics analysis. BioMed Res Int. (2022) 2022:7992981. doi: 10.1155/2022/7992981 36212719 PMC9536999

[42] Akinrinmade OA Chetty S Daramola AK Islam MU Thepen T Barth S. CD64: an attractive immunotherapeutic target for M1-type macrophage mediated chronic inflammatory diseases. Biomedicines. (2017) 5:56. doi: 10.3390/biomedicines5030056 28895912 PMC5618314

[43] Theeuwes WF Di Ceglie I Dorst DN Blom AB Bos DL Vogl T. CD64 as novel molecular imaging marker for the characterization of synovitis in rheumatoid arthritis. Arthritis Res Ther. (2023) 25:158. doi: 10.1186/s13075-023-03147-y 37653557 PMC10468866

[44] di Masi A Ascenzi P. H2S: A “Double face” molecule in health and disease. Biofactors. (2013) 39:186–96. doi: 10.1002/biof.1061 23233276

[45] Morris AA Kožich V Santra S Andria G Ben-Omran TI Chakrapani AB. Guidelines for the diagnosis and management of cystathionine beta-synthase deficiency. J inherit Metab dis. (2017) 40:49–74. doi: 10.1007/s10545-016-9979-0 27778219 PMC5203861

[46] Saha S Chakraborty PK Xiong X Dwivedi SK Mustafi SB Leigh NR. Cystathionine β-synthase regulates endothelial function via protein S-sulfhydration. FASEB J. (2016) 30:441–56. doi: 10.1096/fj.15-278648 PMC4684530 26405298

[47] Song C Zhou D Cheng K Liu F Cai W Mei Y. Bioinformatics-based discovery of intervertebral disc degeneration biomarkers and immune-inflammatory infiltrates. JOR Spine. (2023) 7:e1311. doi: 10.1002/jsp2.1311 38222811 PMC10782055

[48] Sun H Qi L Wang S Li X Li C. Hydrogen sulfide is expressed in the human and the rat cultured nucleus pulposus cells and suppresses apoptosis induced by hypoxia. PloS One. (2018) 13:e0192556. doi: 10.1371/journal.pone.0192556 29466396 PMC5821346

[49] Xu D Jin H Wen J Chen J Chen D Cai N. Hydrogen sulfide protects against endoplasmic reticulum stress and mitochondrial injury in nucleus pulposus cells and ameliorates intervertebral disc degeneration. Pharmacol Res. (2017) 117:357–69. doi: 10.1016/j.phrs.2017.01.005 28087442

[50] Dilek N Papapetropoulos A Toliver-Kinsky T Szabo C. Hydrogen sulfide: An endogenous regulator of the immune system. Pharmacol Res. (2020) 161:105119. doi: 10.1016/j.phrs.2020.105119 32781284

[51] Mariggiò MA Pettini F Fumarulo R. Sulfide influence on polymorphonuclear functions: a possible role for Ca2+ involvement. Immunopharmacol immunotoxicol. (1997) 19:393–404. doi: 10.3109/08923979709046984 9248866

[52] Mariggiò MA Minunno V Riccardi S Santacroce R De Rinaldis P Fumarulo R. Sulfide enhancement of PMN apoptosis. Immunopharmacol immunotoxicol. (1998) 20:399–408. doi: 10.3109/08923979809034822 9736444

[53] Faller S Hausler F Goeft A von Itter MA Gyllenram V Hoetzel A. Hydrogen sulfide limits neutrophil transmigration, inflammation, and oxidative burst in lipopolysaccharide-induced acute lung injury. Sci Rep. (2018) 8:14676. doi: 10.1038/s41598-018-33101-x 30279441 PMC6168479

[54] Yang R Qu C Zhou Y Konkel JE Shi S Liu Y. Hydrogen sulfide promotes tet1- and tet2-mediated foxp3 demethylation to drive regulatory T cell differentiation and maintain immune homeostasis. Immunity. (2015) 43:251–63. doi: 10.1016/j.immuni.2015.07.017 PMC4731232 26275994

[55] Ji WS Xin Y Zhang LF Liu XQ. ALG2 Influences T cell apoptosis by regulating FASLG intracellular transportation. Biochem J. (2020) 477:3105–21. doi: 10.1042/bcj20200028 32766719

[56] Ma CJ Liu X Che L Liu ZH Samartzis D Wang HQ. Stem cell therapies for intervertebral disc degeneration: immune privilege reinforcement by fas/fasL regulating machinery. Curr Stem Cell Res Ther. (2015) 10:285–95. doi: 10.2174/1574888x10666150416114027 25381758

[57] Liu ZH Sun Z Wang HQ Ge J Jiang TS Chen YF. FasL expression on human nucleus pulposus cells contributes to the immune privilege of intervertebral disc by interacting with immunocytes. Int J Med Sci. (2013) 10:1053–60. doi: 10.7150/ijms.6223 PMC3691805 23801893

