# Supplementary figures and images for: Identification of aging-related biomarkers for intervertebral disc degeneration in whole blood samples based on bioinformatics and machine learning
Source: Front Immunol. 2025 Apr 15;16:1565945. doi: 10.3389/fimmu.2025.1565945 (PMC12037391; doi:10.3389/fimmu.2025.1565945)

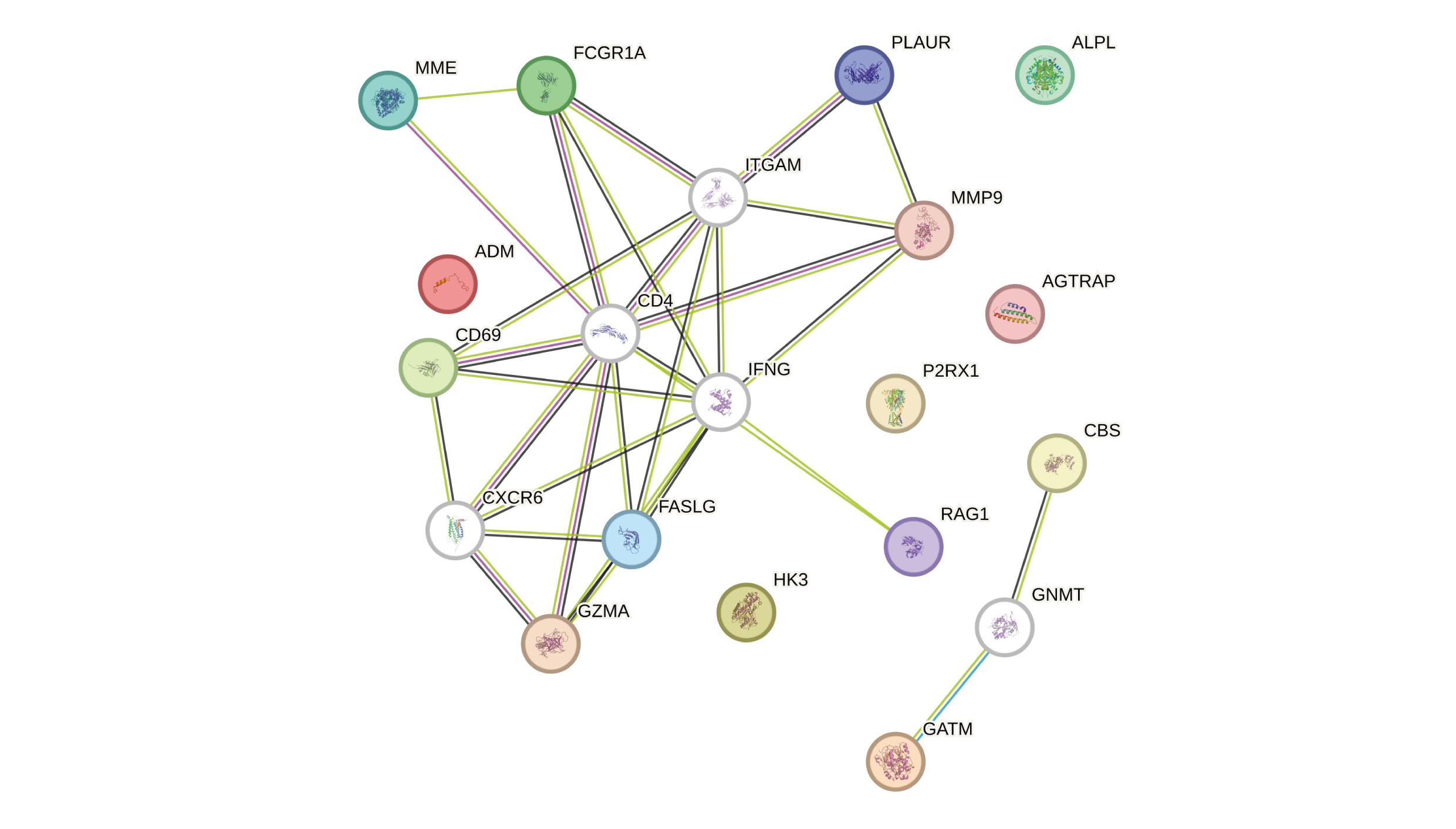

Supplement: Supplementary Figure 1 — PPI network of 15 hub genes. Each node represents a gene, with interactions between them indicated by connecting edges. Nodes are colored based on functional clusters. The interactions are visualized using various edge colors to highlight different types of relationships. [file Image1.tif]

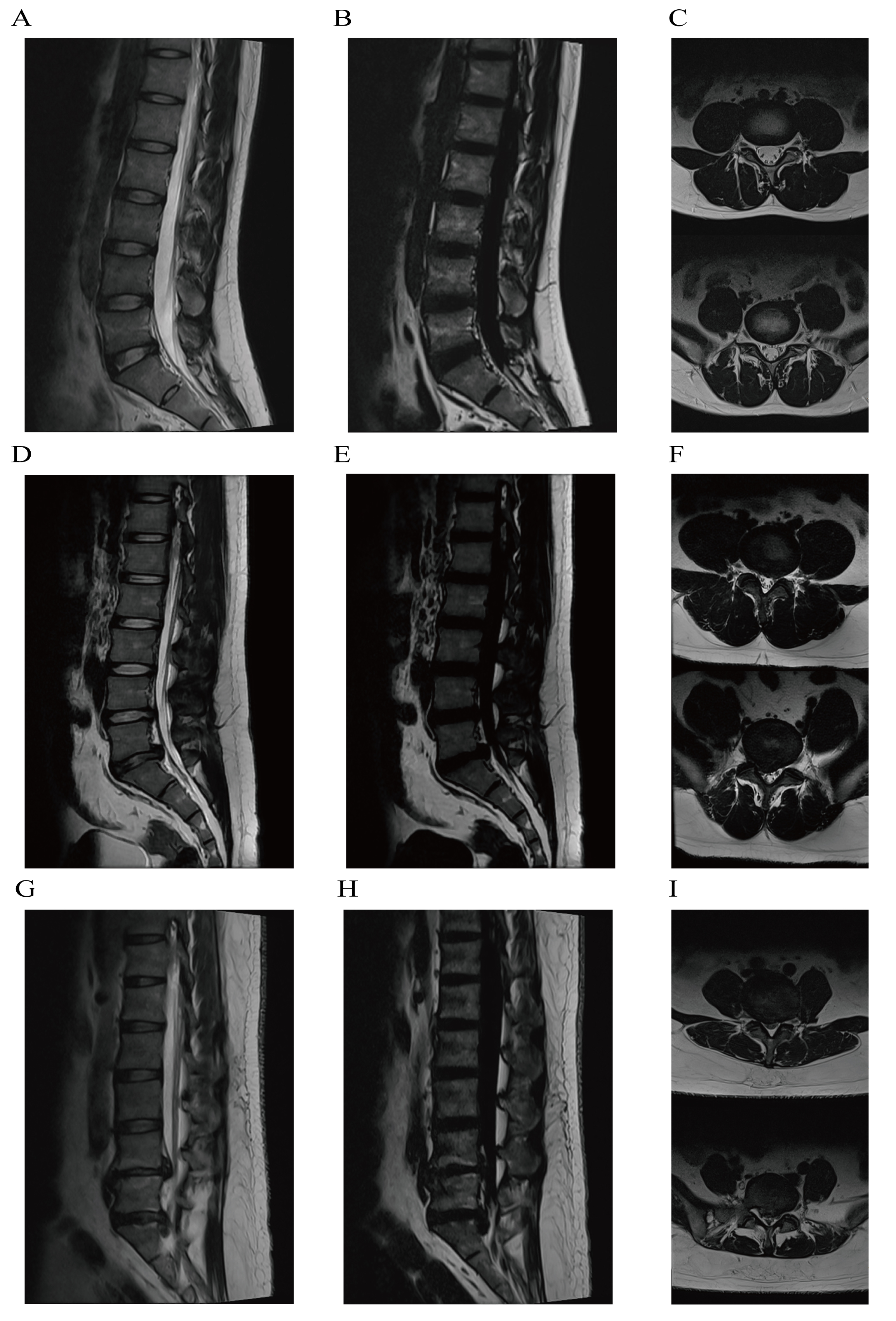

Supplement: Supplementary Figure 2 — Typical MRI findings for different groups: left to right—T2, T1 sagittal, and axial images. (A–C) CON-1, male, 27, Pfirrmann grade 1. (D–F) M-IVDD-2, male, 28, Pfirrmann grade 3. (G–I) S-IVDD-5, female, 40, Pfirrmann grade 4. [file Image2.jpeg]
